# Mechanistic Transformation of CuI Nanoparticles Into Oxidation‐Resistant 2D Copper Nanoplates

**DOI:** 10.1002/smll.202508098

**Published:** 2025-11-18

**Authors:** Hyeuk Jin Han, Moon Young Yang, Changsoo Lee, Gangtae Jin, James L Hart, Rabecca Mutheu, Hyung il Lee, Seo Hyun Kim, Hanhwi Jang, Minjoon Kim, Yeon Sik Jung, William A. Goddard, Judy J. Cha, Chungseok Choi

**Affiliations:** ^1^ Department of Materials Science and Engineering Cornell University Ithaca NY 14853 USA; ^2^ Department of Materials Science and Engineering Sungshin Women's University Seoul 01133 Republic of Korea; ^3^ Materials and Process Simulation Center (MSC) California Institute of Technology Pasadena CA 91125 USA; ^4^ Department of Chemical Engineering Education Chungnam National University 99 Daehak‐ro Daejeon 34134 Republic of Korea; ^5^ Department of Electronic Engineering Gachon University Seongnam 13120 Republic of Korea; ^6^ SKKU Advanced Institute of Nanotechnology and Department of Nanoengineering Sungkyunkwan University Suwon 16419 Republic of Korea; ^7^ Department of Materials Science and Engineering Korea Advanced Institute of Science and Technology 291 Daehak‐Ro, Yuseong‐Gu Daejeon 34141 Republic of Korea

**Keywords:** crystallization mechanism, defect engineering, phase transformation

## Abstract

Unconventional phase transformations reveal new crystallization mechanisms, yet direct observation of such pathways during nanoscale solution‐phase synthesis remains challenging. This study uncovers an atypical growth process in which thermodynamically stable CuI nanoparticles (NPs) transform into high‐energy 2D Cu plates. Using a combination of in situ transmission electron microscopy, ex situ structural analysis, and density functional theory calculations shows that the formation of structural defects induced by hexadecylamine and chloride ions facilitates the transformation by promoting surface iodine vacancies. The resulting Cu{111} nanoplates, with ultrathin thicknesses (≈4 nm) and exceptionally high aspect ratios (≈450), display enhanced oxidation resistance and long‐term stability under ambient conditions. This resistance is attributed to the close‐packed {111} facets, which suppress chemical oxidation even after extended exposure to air over 100 days. These findings provide new insights into non‐classical crystallization pathways in metal nanomaterials and suggest a versatile approach for preparing oxidation‐resistant, structurally defined Cu nanostructures.

## Graphical abstract

Thermodynamically stable CuI nanoparticles transform into less stable 2D Cu plates through a defect‐mediated uphill phase transformation. In situ TEM and DFT analyses reveal that Cl^−^ and hexadecylamine promote iodine vacancies, enabling Cu aggregation and facet‐selective {111} growth. The resulting ultrathin Cu plates exhibit remarkable oxidation resistance and long‐term structural stability under ambient conditions.

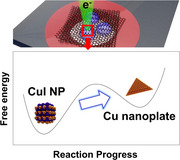

## 1 Introduction

Copper (Cu) has long been a key material in electronics and thermal systems due to its high electrical conductivity (6.2 × 10⁷ S m^−1^), thermal conductivity (386 W m^−1^ K^−1^), and ductility.^[^ ^1^, ^2^ ^]^ Its abundance makes it a cost‐effective alternative to noble metals such as Ag, Au, and Pt.^[^ ^3^ ^]^ At the nanoscale, Cu exhibits distinct properties: for example, Cu nanowire (NW) arrays transmit up to 91% of incident light,^[^ ^4^ ^]^ unlike reflective bulk Cu, and sustain elastic strains up to ≈8% with a *Young's* modulus of ≈80 GPa, four times higher than bulk Cu.^[^ ^5^, ^6^ ^]^ These unique features have driven interest in synthesizing Cu nanomaterials with controlled size and shape.^[^ ^7^, ^8^, ^9^, ^10^ ^]^ Despite these advancements, the practical deployment of Cu nanostructures is severely limited by their high susceptibility to oxidation, driven by their elevated surface energies. This oxidation significantly compromises their electrical and optical performance.^[^ ^7^ ^]^ Furthermore, the precise control of crystallization processes necessary for achieving structurally defined and oxidation‐resistant Cu nanocrystals remains elusive due to incomplete mechanistic understanding.

**Figure 1:**
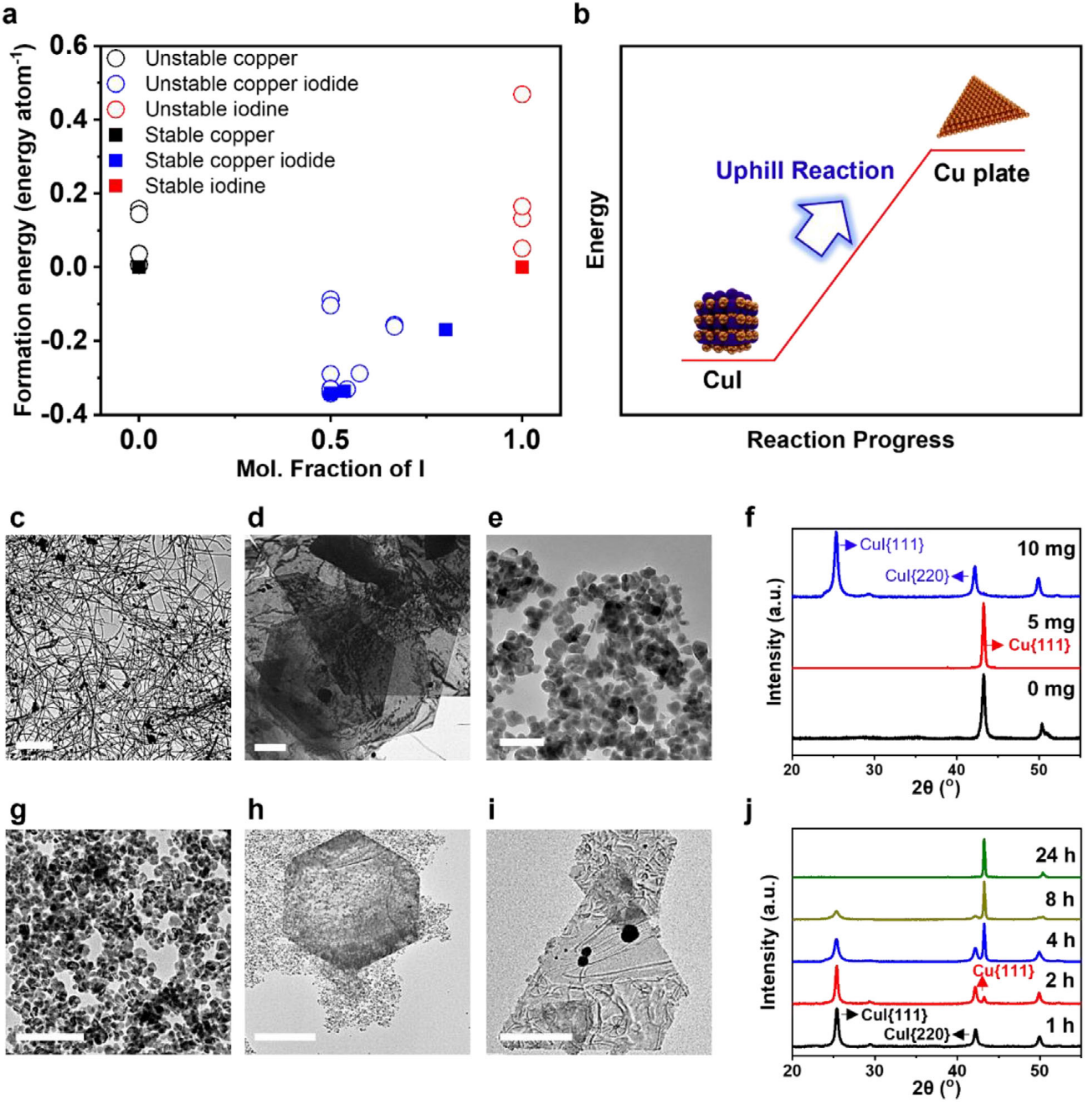
a) The formation energy of CuI and Cu. The thermodynamic data was adapted from Materials Projects.^[^ ^20^ ^]^ b) Uphill reaction from CuI NPs to Cu plate. c) TEM image of Cu NWs obtained without iodine after 8 h. Scale bar, 1 µm. d) TEM image of 2D Cu plates synthesized with 5 mg iodine after 8 h. Scale bar, 2 µm. e) TEM image of CuI NPs synthesized with 10 mg iodine after 8 h. Scale bar, 500 nm. f) PXRD patterns of samples obtained with 0 mg (Cu NWs), 5 mg (Cu plates), and 10 mg (CuI NPs) iodine, respectively. g–i) TEM images showing time‐dependent evolution with 10 mg iodine: (g) CuI NPs after 4 h (Scale bar, 200 nm); (h) mixed CuI NPs and Cu plates after 8 h (Scale bar, 2 µm); (i) fully transformed Cu plates after 24 h (Scale bar, 2 µm). j) PXRD patterns of samples obtained with 10 mg iodine at different reaction times (4, 8, 24 h).

Although recent studies have demonstrated the synthesis of Cu nanoplates using iodine,^[^ ^10^, ^11^ ^]^ yet the fundamental crystallization mechanisms remain poorly understood, especially regarding transformations involving unusual thermodynamic pathways. Typically, Cu–I compound formation dominates due to the high reduction potentials (e.g., Cu^2^⁺ + I^−^ + e^−^ → CuI, E° = 0.86 V vs SHE),^[^ ^12^ ^]^ the very low solubility of CuI in aqueous media,^[^ ^13^ ^]^ and strong thermodynamic stability (**Figure** **1**a).^[^ ^14^ ^]^ This dominance of CuI formation greatly complicates direct synthesis routes to metallic Cu in aqueous media. Therefore, clearly understanding how metallic Cu can be preferentially formed under iodine‐rich conditions requires examining crystallization via two distinct yet complementary frameworks: redox crystallization and classical crystallization.

In the initial stage, redox crystallization governs cluster formation and nucleation. Compared to Cu (Cu^2^⁺ + 2e^−^ → Cu, E° = 0.34 V; Cu_2_O + H_2_O + 2e^−^ → 2Cu + 2OH^−^, E° = 0.47 V), CuI has a much higher reduction potential and limited solubility in aqueous media, making its conversion to Cu rarely observed.^[^ ^15^, ^16^ ^]^ Once CuI nuclei are formed, growth typically follows the classical principle of free‐energy minimization, favoring stable final phases. Thus, the transformation from stable CuI to Cu is considered thermodynamically unfavorable, as illustrated in Figure 1a,b.^[^ ^16^, ^17^ ^]^

In this work, we elucidate an unconventional uphill transformation pathway where stable CuI nanoparticles spontaneously transform into higher energy metallic Cu nanoplates with an ultrathin thickness (≈4 nm) and a high aspect ratio (≈450). Despite the stability of CuI under ambient conditions and its persistence in the solid phase across a wide temperature range,^[^ ^16^, ^18^, ^19^ ^]^ metallic Cu emerges as the final product. In situ transmission electron microscopy (TEM) observations directly visualize the transformation process, revealing the structural evolution occurring at the nanoscale. Density functional theory (DFT) calculations provide complementary insights, indicating that the transformation is driven by structural defects induced by the presence of hexadecylamine (HDA) and Cl^−^ ions. Remarkably, the resulting Cu{111} nanoplates exhibit exceptional oxidation resistance, directly addressing the major challenge that previously hindered the practical applicability of Cu nanostructures. These findings provide mechanistic insights into non‐classical crystallization pathways at the nanoscale and suggest a new approach for realizing oxidation‐resistant, structurally tailored Cu nanomaterials.

## 2 Results

### 2.1 Synthesis and Structural Characterization of Cu Nanomaterials

Both 2D Cu plates and CuI nanoparticles were synthesized by modifying the Cu NW protocol (Figure 1c), with the outcome determined by the amount of iodine added (see Methods for details).^[^ ^21^ ^]^ A higher iodine concentration (10 mg in 10 mL water) led to CuI NPs, whereas a lower concentration (5 mg) yielded 2D Cu plates. This trend is consistent with previous findings that CuI dominates under higher I^−^ concentrations.^[^ ^16^ ^]^

**Figure 2:**
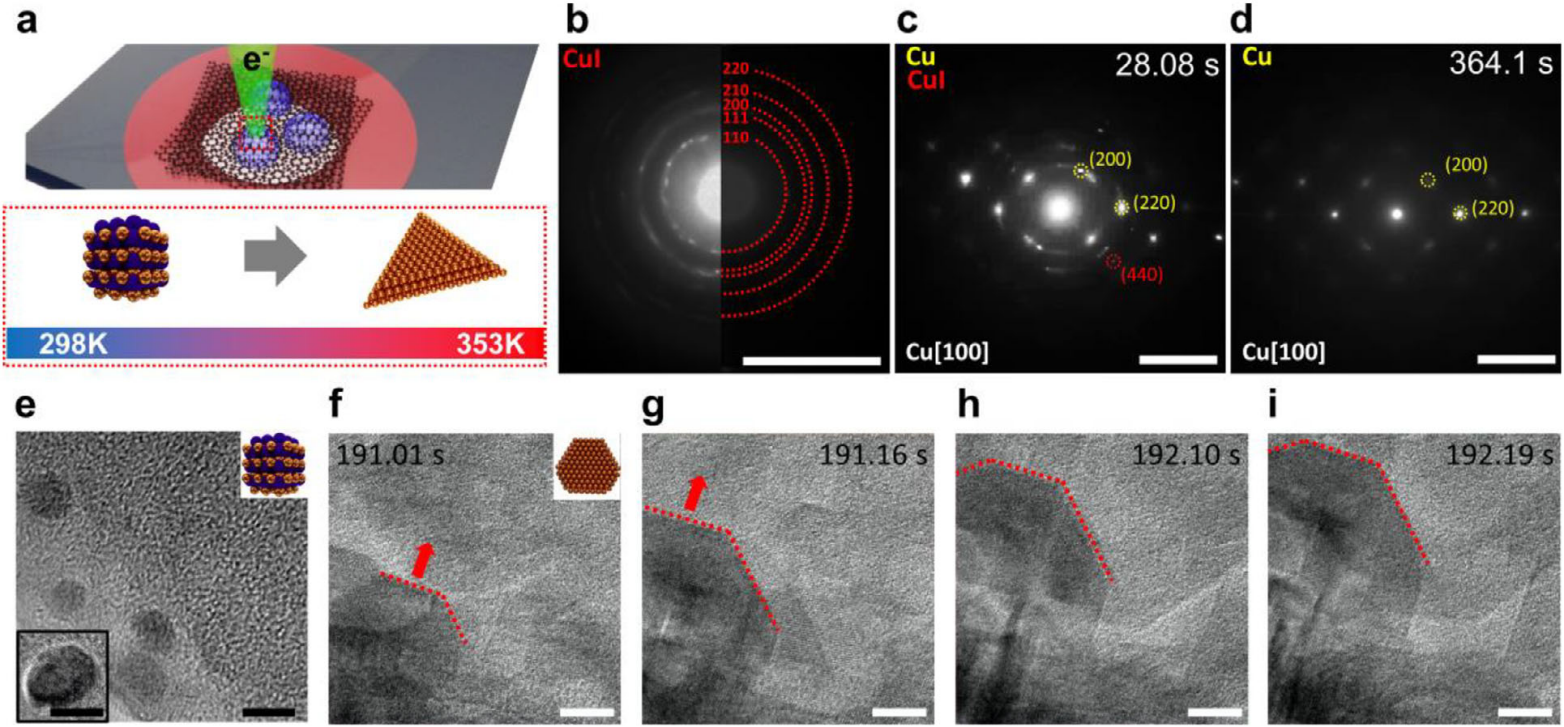
a) Schematic image of in situ graphene liquid cell TEM to investigate synthesis of 2D Cu plates from CuI NPs upon heating. b) SAED pattern of initial CuI NPs in graphene liquid cell before heating. Scale bar, 5 1/nm. c,d) Snapshots from an in situ movie in SAED mode with heating (Movie S1, Supporting Information). (c) shows a mixed CuI and Cu pattern in the initial stage, whereas (d) shows only a single crystal Cu SAED pattern. Scale bars, 10 1/nm. e) TEM image of initial CuI NPs encapsulated in the graphene liquid cell before heating. Scale bars, 5 nm. f–i) TEM images from the in situ TEM movie (Movie S2, Supporting Information), showing the growth of 2D Cu nanoplates at 80 °C. Scale bars, 5 nm.

We conducted powder X‐ray diffraction (PXRD) and TEM to characterize the synthesized nanomaterials. TEM showed the presence of Cu NWs without iodine molecules, with PXRD peaks that match the Cu JCPDS (#00‐004‐0836), as shown in Figure 1c,f. When iodine was introduced at varying concentrations, the morphology evolved from Cu NWs (Figure 1c) to 2D Cu plates (Figure 1d) and eventually to CuI NPs (Figure 1e). The synthesized Cu plates are 2D structures with large lateral dimensions (Figure S1, Supporting Information). Regions consisting of only a few atomic layers appear transparent in TEM, STEM and SEM images. Side view SEM images revealed plates thinner than 8 nm (Figure S1e,f, Supporting Information). Atomic force microscopy (AFM) further confirmed the thickness values, with a representative thickness of ≈4 nm (Figure S2a, Supporting Information). Across multiple samples, the overall thickness distribution of the Cu plates ranges from ≈5 to 50 nm (Figure S2, Supporting Information).

PXRD analysis of the 2D Cu plates revealed a strong Cu{111} peak with negligible Cu{200} reflection (Figure 1f, red). Lattice‐resolved TEM confirmed a {111}‐oriented surface (Figure S3, Supporting Information), indicating facet‐selective growth. Under 10 mg iodine conditions, CuI nanoparticles emerged within 1 h, converted into 2D Cu plates over 2–8 h, and fully transformed by 24 h (Figure 1g–j). In contrast, at lower iodine concentrations (5 mg), weak CuI peaks were detected at 4 h by both PXRD and energy‐dispersive X‐ray spectroscopy (EDX), indicating that some CuI nanoparticles formed transiently during the reaction (Figure S4, Supporting Information). However, after 8 h Cu plates had fully formed, and no CuI was detectable. In addition, time‐sequenced TEM images illustrating the complete conversion from CuI nanoparticles to 2D Cu plates are provided in Figure S5 (Supporting Information), which clearly reveal the progressive morphological and lattice evolution across the three stages: pristine CuI nanoparticles, mixed CuI–Cu intermediates, and fully formed Cu plates. These results point to an unusual, non‐classical crystallization pathway in which stable CuI nanoparticles transform into metastable 2D Cu plates, a process likely overlooked in earlier studies due to rapid transformation kinetics.^[^ ^10^, ^16^ ^]^ This uphill phase transformation from stable CuI NPs to metastable 2D Cu plates deviates from classical crystallization pathways. It likely proceeds via phase decomposition and plays a critical role in forming ultrathin Cu structures.

### 2.2 In Situ TEM Observation of the CuI‐To‐Cu Plate Transformation

To investigate the origin of the 2D Cu plates and the underlying growth mechanism in solution, we conducted in situ liquid cell TEM experiments with heating. **Figure** **2**a illustrates the graphene based liquid cell, composed of two graphene membranes encapsulating a CuI NP solution on a heating compatible TEM grid (see Methods for details). Using high resolution TEM, we monitored the structural evolution via bright field (BF) imaging and selected area electron diffraction (SAED).

Before heating, BF TEM and SAED confirmed the presence of CuI NPs (Figure 2b,e). Upon heating the cell to 80 °C, we recorded in situ SAED mode movies (Movie S1, Supporting Information) and observed the nucleation and growth of 2D Cu plates. The representative SAED snapshots corresponding to the overall phase evolution captured in Movie S1 (Supporting Information) are shown in Figure 2c,d. The detailed movie sequence is provided in Figure S6 (Supporting Information) together with dynamic timestamps for temporal reference. Time resolved SAED patterns revealed the coexistence of CuI and Cu phases shortly after heating (Figure 2c), followed by complete transformation to single crystalline Cu (Figure 2d). Representative TEM snapshots captured the morphological evolution of nanosheet formation at 80 °C, as observed in Movie S2 (Supporting Information), are shown in Figure 2e–i, while the corresponding movie sequence with dynamic timestamps is presented in Figure S7 (Supporting Information). The disappearance of spherical CuI NPs and concurrent growth of the Cu(220) facet were clearly observed, consistent with ex situ PXRD results (Figure 1j) and in situ SAED (Figure 2c,d). Control experiments confirmed that no phase transformation occurred under electron beam irradiation alone (Figure S8 and Movie S3, Supporting Information), ruling out beam induced effects.

**Figure 3:**
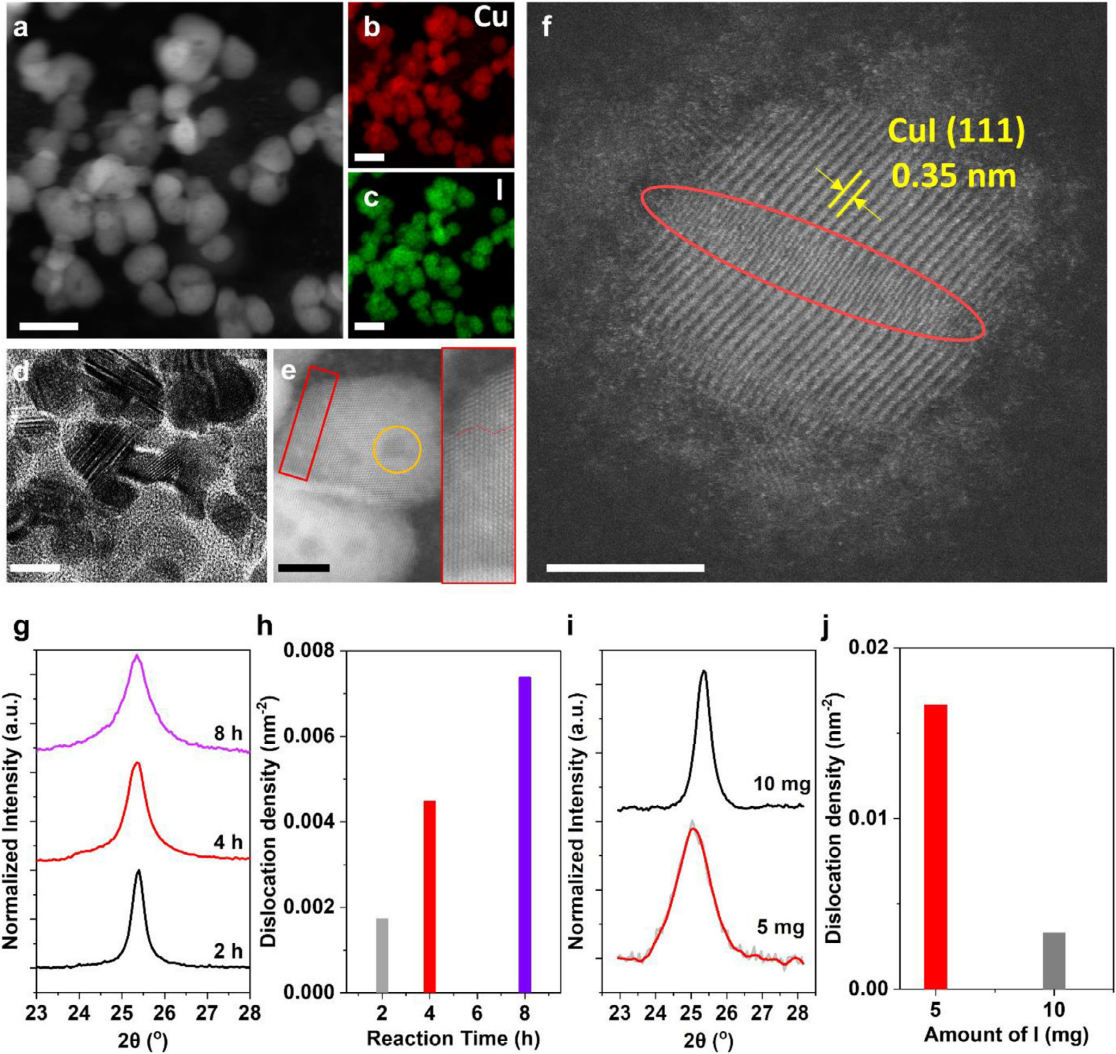
a) High‐angle annular dark‐field STEM (HAADF‐STEM) image of CuI NPs and EDX mapping of b) Cu and c) I of the same imaging region. Scale bars, 50 nm. d–f) TEM and HAADF‐STEM images of CuI NPs with defects with addition of 5 mg iodine. Scale bars, 20 nm (d), 10 nm (e), and 5 nm (f). g) PXRD of CuI{111} by reaction time with 10 mg KI. h) Calculated defect density from (g). i) PXRD of CuI{111} by iodine amount at 8 h reaction. j) Calculated defect density from (i).

Scanning transmission electron microscopy (STEM)–EDX mapping confirmed the morphology and composition of representative CuI NPs (**Figure** **3**a–c). High resolution STEM images revealed dense structural defects, including multiple twin boundaries (Figure 3d, red box in Figure 3e), and darker regions corresponding to nanoscale voids or surface pits (yellow circle in Figure 3e). In Figure 3f, a CuI NP shows a defect region running through the center (red circle), where the lattice spacing (≈3.5 Å) matches the CuI(111) planes in undistorted regions, while the defective region exhibits doubled lattice periodicity. This contrast is consistent with a dislocation where both the line and Burgers vectors have in plane components, resulting in a half plane offset across the defect core.^[^ ^22^, ^23^ ^]^ STEM image simulations further support this interpretation (Figure S9, Supporting Information), though other defect types may also contribute.

### 2.3 Mechanistic Insights of Defect‐Mediated Phase Transformation in CuI NPs

To quantify the structural disorder in CuI NPs, we calculated defect densities based on PXRD peak broadening and shifts (see Methods). Samples reacted for 8 h exhibited broader and shifted peaks (Figure 3g,i), indicating higher defect concentrations than those reacted for 2 h. The corresponding defect density was approximately four times greater after 8 h (Figure 3h), suggesting that prolonged reaction time increases defect accumulation. Similarly, reducing iodine concentration also led to a substantial increase in peak broadening (Figure 3i) implies an increase in defect density (Figure 3j), underscoring the importance of iodine concentration in determining crystallinity. Collectively, both extended reaction time and lower iodine concentration promote defect formation, which may facilitate the transformation from CuI nanoparticles to 2D Cu plates.

Based on the observed voids and crystallographic defects (Figure 3d–f), we hypothesize that these defect sites act as nucleation centers for metallic Cu, triggering the atypical phase transformation. To validate this hypothesis, we performed control experiments using commercial CuI NPs (Sigma–Aldrich) and DFT calculations. To test this hypothesis, we performed control experiments using commercial CuI nanoparticles under conditions replicating the original synthesis but omitting the initial Cu nanowire step (see Methods).

**Figure 4:**
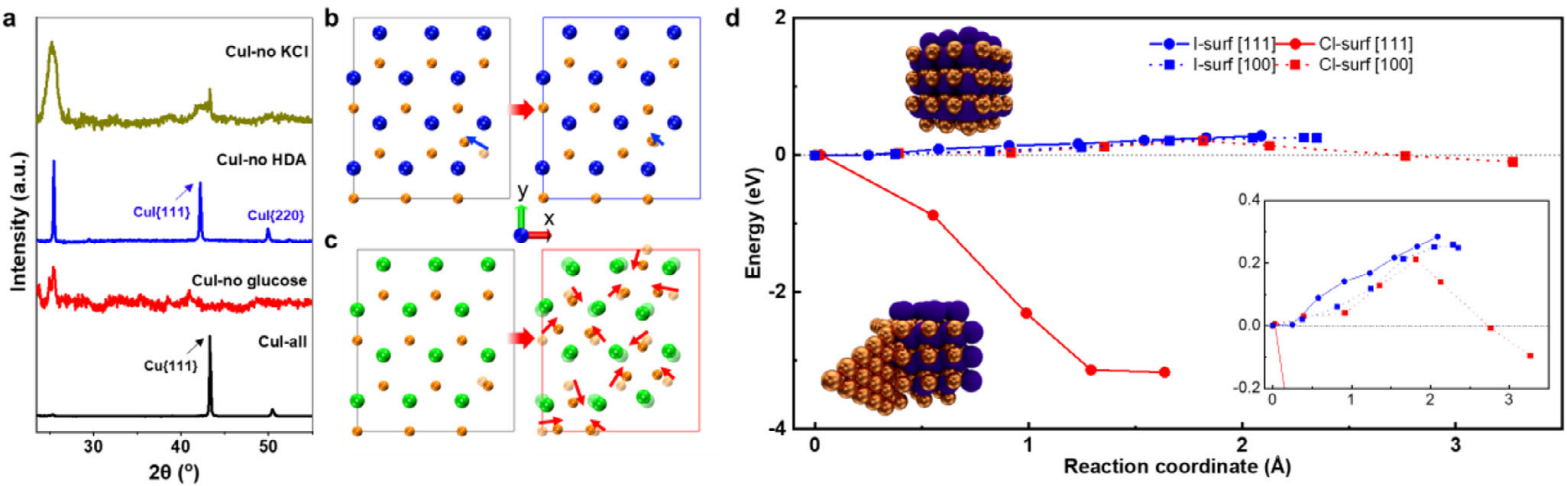
a) PXRD of commercial CuI NPs after reactions at 100 C° for 8 h with different chemical combinations. b,c) Surface rearrangement by single Cu atom movement on (b) the I‐terminated [111] surface and (c) the Cl‐terminated [111] surface from DFT calculations, where the initial positions are shown with transparency and only top two layers are shown for clarity. Blue, orange, and green represent I, Cu, and Cl, respectively. d) The energy profile along the single Cu atom movement on the surface at different surface conditions. The inset shows a zoomed‐in energy range from −0.2 to 0.4 eV.

PXRD and TEM analyses confirmed that, when glucose, HDA, and Cl^−^ ions were present, the direct phase transformation from CuI to metallic Cu plates occurred (**Figure** **4**a; Figure S10, Supporting Information). Notably, omitting HDA led to PXRD patterns characteristic of pristine CuI NPs, confirming that HDA is essential for the transformation (Figure 4a). Similarly, in the absence of glucose, no metallic Cu peaks were detected, supporting its role as the reducing agent. In control experiments without KCl, the reaction mixture evaporated during heating, and only CuI and Cu phases—without 2D plate formation—were observed by TEM (Figure S10, Supporting Information). These results further confirm the synergistic roles of HDA and Cl^−^ ions in facilitating CuI decomposition, with glucose enabling the reduction of Cu⁺ to metallic Cu.

### 2.4 DFT Analysis of Iodine Vacancy Formation and Surface Restructuring in CuI NPs

To understand the mechanistic origin of this transformation, we conducted DFT calculations at the PBE‐D3 level. Surface energy analysis showed that CuI surfaces are iodine‐terminated for both {111} and {100} facets (Figure S11 and Table S1, Supporting Information).^[^ ^24^ ^]^ Given HDA's high pKa (≈10.6),^[^ ^25^ ^]^ it is positively charged in solution and likely interacts electrostatically with surface iodide anions. This interaction may lead to surface iodine vacancy formation, exposing underlying Cu and enabling the transformation. However, calculated defect formation energies for iodine removal by HDA alone were high (1.154 eV for {111}, 0.678 eV for {100}), suggesting the process is energetically unfavorable. In contrast, when Cl^−^ ions are present, the formation energies become negative (−0.044 eV for {111}, −0.226 eV for {100}), indicating a favorable reaction pathway. These results suggest that Cl^−^ stabilizes iodine vacancy formation by passivating the defect sites, enabling the phase transformation. This is consistent with our experimental observations of defective, pit rich CuI NPs (Figure 3) and the critical role of Cl^−^ in the control reactions (Figure 4a,b).

For the phase transformation from CuI NPs to Cu plates, Cu ions must migrate and aggregate to form the metallic phase. To explore this process, we performed DFT calculations simulating the movement of a surface Cu ion toward a neighboring Cu site on CuI surfaces. This aimed to assess whether subtle surface perturbations, potentially induced by solvent interactions at elevated temperatures, could trigger surface rearrangement and Cu aggregation. On the I‐terminated {111} surface, a displacement of at least 2 Å was required to bring a Cu ion close to its neighbor; smaller displacements led to relaxation back to the original position (Figure 4c, left). Even with greater displacement, the overall surface remained unchanged (Figure 4c, right), and the process was energetically unfavorable (Figure 4e). Similar results were obtained for the I‐terminated {100} surface and confirmed that Cu aggregation is unlikely on pristine CuI surfaces (Figure 4e; Figure S12a, Supporting Information).

We next modeled Cl‐terminated CuI surfaces, assuming complete substitution of surface iodide by Cl^−^ (Figure S13, Supporting Information), a thermodynamically favorable scenario in the presence of HDA. On the Cl‐terminated {111} surface, a small displacement of a single Cu ion (≈0.5 Å) induced a cooperative rearrangement of surface Cu atoms, resulting in significant aggregation (Figure 4d). This transformation was energetically favorable and proceeded with almost no energy barrier (Figure 4e). Notably, this behavior was unique to the {111} facet. On the Cl‐terminated {100} surface, even larger displacements (>3 Å) failed to initiate surface rearrangement, despite a slightly more favorable final energy (Figure 4e, Figure S12b, Supporting Information).

This facet‐selective behavior is attributed to the interlayer distances between Cu and halide atoms. In pristine CuI, the Cu–I interlayer distances are 0.75 and 1.36 Å for the {111} and {100} surfaces, respectively. Upon Cl termination, these distances decrease to 0.34 and 0.79 Å, respectively, due to the smaller ionic radius of Cl^−^ (Figures S11 and S13, Supporting Information). The reduced spacing on the {111} facet leads to near surface exposed Cu ions, facilitating aggregation and growth into Cu plates.

These computational findings are consistent with experimental observations: voids and pits in high‐angle annular dark field STEM (HAADF‐STEM) images, increased defect density in PXRD during prolonged reactions, and the critical role of HDA and Cl^−^ ions in facilitating the transformation. Together, the DFT results elucidate how Cl^−^‐mediated surface restructuring enables the phase decomposition of CuI NPs and drives the formation of 2D Cu plates with exposed {111} facets.

### 2.5 Oxidation Resistance and Electronic Properties of 2D Cu Plates

**Figure 5:**
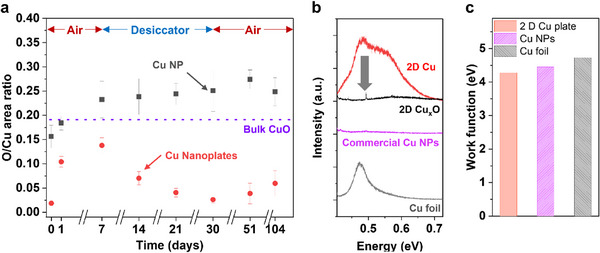
a) Oxidation resistance of 2D Cu plates and commercial Cu NPs as a function of storage time. b) PL of Cu materials by different structure and phase. c) Work function of Cu materials by different structures.

Notably, the 2D Cu plates exhibited exceptional oxidation resistance. We monitored oxidation by tracking Cu 2p and O 1s peaks using X‐ray photoelectron spectroscopy (XPS) over ≈100 days, in comparison with commercial Cu NPs (Figure S14, Supporting Information). Samples were prepared in an Ar filled glove box and transferred to the XPS chamber without air exposure. After establishing initial oxidation states, the samples were exposed to ambient air. After 1 day, Cu plates showed increased surface oxygen but minimal chemical oxidation. By 7 days, only slow oxidation was observed (**Figure** **5**a), whereas Cu NPs exhibited rapid oxidation from day 1 and continued degradation through day 7.

Subsequent storage in a desiccator (<10% RH) for 7 days (total of 14 days) further differentiated the materials. Cu NPs retained high oxygen content, even exceeding that of bulk CuO, while Cu plates showed a significant reduction in oxygen signal that remained low for 30 days. Upon re‐exposure to ambient air, the Cu plates remained stable for up to 105 days. These results suggest that much of the detected oxygen was physisorbed and not chemically bond. The oxidation resistance is attributed to the close‐packed Cu{111} surface, which provides enhanced stability against ambient oxidation.^[^ ^26^ ^]^

The preserved metallic state of the Cu plates was confirmed by photoluminescence (PL) spectroscopy (Figure 5b). Commercial Cu foil showed a characteristic PL emission at 2.137 eV (580 nm), consistent with bulk Cu.^[^ ^27^ ^]^ Although PL measurements on isolated Cu plates were difficult due to their optical transparency, ensemble measurements revealed a broad PL spectrum, likely reflecting thickness variations. The plates exhibited a reduced work function (≈4.2 eV) compared to bulk Cu foil (≈4.7 eV) (Figure 5c), attributed to quantum confinement effects. The finite thickness induces band quantization near the Fermi level, resulting in PL redshift due to transitions from quantized states below the Fermi level to holes in the d‐band.^[^ ^28^ ^]^

Upon thermal oxidation at 120 °C for 30 min, the Cu plates lost their PL signal, consistent with the oxidation sensitivity of metallic Cu. Similarly, oxidized Cu NPs exhibited no PL response. These results demonstrate that surface oxidation suppresses bulk‐like optical properties in Cu nanomaterials, whereas 2D Cu plates retain their electronic features under ambient conditions. This oxidation resistance, combined with quantum confinement, offers a promising route for tuning the properties of metallic Cu through dimensional control.

To further demonstrate the applicability of the synthesized Cu nanoplates, we also performed preliminary SERS measurements using the 2D Cu plates as substrates (Figure S15, Supporting Information). The plates exhibited moderate Raman signal enhancement compared to non‐SERS‐active substrates, indicating their feasibility as SERS platforms, although further optimization will be required.

## 3 Conclusion

This work demonstrates an unconventional uphill phase transformation from stable CuI NP to 2D Cu nanoplates exhibiting enhanced oxidation resistance. In situ TEM directly visualized the dynamics of this transformation, while ex situ TEM and X‐ray diffraction (XRD) confirmed the evolution of phases that depart from classical crystallization theory. Comprehensive experimental and DFT analyses show that surface iodine vacancies, promoted by the presence of HDA and Cl^−^ ions, are essential for initiating the formation of metallic Cu plates. The resulting 2D Cu nanostructures exhibit superior oxidation resistance, which is attributed to the prevalence of close‐packed Cu{111} facets. This finding not only advances our understanding of metastable phase formation during solution‐phase synthesis, but also introduces a promising approach for the synthesis of stable, dimensionally confined Cu nanomaterials. Such 2D Cu plates may enable new opportunities for catalytic applications, low‐resistivity interconnects, transparent conductors, and other advanced electronic devices.

## 4 Experimental Section

### Chemicals

Copper(II) chloride dihydrate (CuCl_2_·2H_2_O, 99.999%), D‐(+)‐Glucose (> 99.5%), Hexadecylamine(HDA)(> 98%), Ethanol (200 proof), 25 nm Cu NPs, KI, Iodine were purchased from Sigma–Aldrich. Hexane (99.9%) were purchased from Fisher Chemical. Deionized water (18.2 MΩ cm at 25 °C) from a Millipore water purification system was used in water solutions. All commercial chemicals were used without additional purification.

### Preparation of Cu NWs or 2D Plates

CuCl_2_·6H_2_O of 22 mg, 50 mg D‐(+)‐Glucose, and 180 mg HDA were dissolved in 10 mL DI water in 30 mL vial, and mixed them using sonication for 15 min. The vial with the mixture solution was transferred to an oil bath. The temperature of oil bath was heated to 100 °C and kept it for 8 h, and finally cooled it to room temperature. The hexane/ethanol (1:1 volume) solvent was used to wash the synthesized Cu NWs five times with sonication for 20 min. The supernatants of synthesized materials were collected by centrifuge. For the synthesis of 2D plates, 60 mM KI solution was prepared and added 1 or 0.5 mL of the KI solution to the mixture solution for Cu NWs preparation. 5 mg or 10 mg iodine were weighted and put in 10 mL water, then this study used only dissolved iodine in water.

### Proving Chemical Roles in Phase Transformation From CuI NPs to 2D Cu Plates

CuI NPs were used from Sigma Aldrich to confirm chemical roles in phase transformation from CuI NPs to 2D Cu plates. 4.2 mg CuI NPs were added in 3.2 mg KCl, 50 mg D‐(+)‐Glucose, and 180 mg HDA in 10 mL DI water in 30 mL vial, and mixed in sonication for 15 min. The vial was put in an oil bath, increased the temperature to 100 °C, and kept it for 8 h. After the synthesized solution was cooled to room temperature, it was washed with the hexane/ethanol (1:1 volume) solvent and collected by centrifuge. For control experiments, one of the chemicals in mixtures was removed systematically, which provided each chemical's contribution to 2D Cu growth.

### Materials Characterizations

Structural characterizations of the nanomaterials were carried out using a SEM and TEM. A field emission SEM (Hitachi S‐4800) was used at an acceleration voltage of 10 kV and a working distance of 5 mm. TEM and STEM images were obtained using a FEI CM120 TEM at 120 kV, a FEI Talos F200X at 200 kV, and an aberration‐corrected ThermoFisher Titan TEM operating at 300 kV. For chemical compositions of the samples XPS data were acquired using a PHI VersaProbe II X‐ray photoelectron spectrometer with an Al Kα target (1486.7 eV). The XRD measurements were carried out using a multipurpose thin‐film X‐ray diffractometer (D/Max 2500; Rigaku). The thicknesses of the Cu plates were determined by a Cypher ES atomic force microscope (AFM) from Asylum Research, and Bruker Dimension Fastscan AFM. PL spectra (Princeton Instruments SP 2300i) were collected with 375 nm diode laser (Picoquant, LDH‐D‐C‐375).

### Work Function Analysis

Ultraviolet photoelectron spectroscopy (UPS) was conducted using Kratos AXIS Ultra DLD spectrometer to analyze the work function of Cu materials. He I (hv = −21.22 eV) was used to scan the kinetic energy of Cu materials. Negative potentials of −5V were applied to measure emitted electrons with maximum kinetic energy. To check He I energy, the UPS with Cu foil was calibrated, and checked the measured work function with a known Cu work function (4.8 eV). The work function is given by.^[^ ^1^ ^]^

(1) $$e\Phi_{m}=hv-\left(E_{K.max}^{meas}-E_{K.min}^{meas}\right)$$

 *e*Φ_*m*_ is a work function.

 *hv* is the energy of He I.

$E_{K.max}^{meas}$ is the maximum kinetic energy in the spectrum.

$E_{K.min}^{meas}$ is the minimum kinetic energy.

### Quantifying Cu and O From XPS Peaks

XPS samples of synthesized 2D Cu plates and commercial Cu NPs (Sigma–Aldrich) were prepared in Ar Ar‐filled glove box. The Cu samples (2D Cu plates and Cu NPs) were dispersed in anhydrate ethanol, which was drop casted and dried on 1 cm^2^ Si wafer.^[^ ^2^ ^]^ The prepared Cu samples on Si wafers were transferred to XPS chamber through an XPS sample vessel without air exposure. The chemical compositions of Cu samples were quantified by comparing the normalized peak areas using CasaXPS software. Five points for each Cu sample were scanned, and averaged quantified oxygen and copper. Bulk CuO [Copper(II) oxide, 99.99%; Sigma–Aldrich] was used for reference (see detailed analysis of XPS 2p and O 1s peaks in Figure S14, Supporting Information).

### Defect Density From XRD

Scherrer equation was used to calculate defect density. After background removal, full width at half maximum (FWHM) was fitted using Origin software.

(2) $$D=\frac{K\lambda }{\beta cos\theta }$$

D is the mean size of crystalline domains.

K is a dimensionless shape factor of 0.94 for cubic symmetry.

λ is the X‐ray wavelength of 1.5406 (Å).

θ is Bragg angle in radian.

β is FWHM in radian.

The calculated mean size of crystalline domains (D) to defect density was converted with below equation.

(3) $$\delta =\frac{1}{D^{2}}$$

δ is defect density.

### In Situ Liquid TEM and Heating Experiments

All TEM experiments were performed with a FEI Tecnai Osiris 200 kV TEM. A graphene membrane was transferred to an in situ TEM heating grid. Subsequently, a graphene flake covered with PMMA was floated on the reactant solution surface and scooped up with the in situ TEM grid that already had the first graphene membrane, and the sample was then dried. The PMMA was removed in acetone to obtain a graphene liquid cell for in situ TEM heating experiments. This study used commercially available in situ TEM heating grids (E‐FHDS‐VO‐10) and a heating holder (Aduro 300DT System) manufactured by Protochips, Inc. The temperature profile of each substrate was pre‐calibrated by the manufacturer. After attaching the liquid cell to the grid, the grid was heated with a ramp rate of 0.67 °C s^−1^ to reach 80 °C. During the heating, CuI NPs and Cu plates were monitored in real time under the BF TEM imaging mode or SAED mode, which were simultaneously video‐recorded (Snagit software).

### Computational Details

The calculations were performed using the DFT method with the Vienna ab initio simulation package (VASP).^[^ ^3^ ^]^ The projector augmented wave method was used to account for core‐valence interactions. The electronic wave functions were represented by plane‐wave basis set with a cut‐off energy of 500 eV. The generalized gradient approximation (GGA) was used in the form of the Perdew–Burke–Ernzerhof functional for the exchange‐correlation interactions.^[^ ^4^ ^]^ The D3 correction for London Dispersion was used for van der Waals interactions.^[^ ^5^ ^]^ The reciprocal space was sampled by the Monkhorst–Pack scheme with a grid of 2 × 2 × 1 for both CuI{111) and CuI{100} models. The convergence criteria were 1 × 10^−5^ eV energy differences to solve the electronic wave function, and all the geometries were converged to within 0.01 eV energy differences. The energy profiles for Cu migration were calculated by the climbing‐image nudged elastic band method.^[^ ^6^ ^]^ After the geometry optimization using VASP, the solvation effect was treated implicitly using CANDLE solvation with Poisson–Boltzmann model for ionic screening as implemented in JDFTx.^[^ ^7^, ^8^ ^]^

The CuI systems were described by four layers of 2 × 2 supercell with > 20 Å vacuum, where the two bottom layers were fixed.

The surface free energy was defined as follows.^[^ ^9^ ^]^

(4) $$F=\frac{1}{2A}\left(E_{slab}-\sum_{i}n_{i}\mu_{i}\right)$$

where *E*_slab_ is the total energy of the slab, A is the surface area of the slab, and *n_i_* and *µ_i_* are the number and chemical potential of atom *i*.

The chemical potentials of Cu and I atoms were limited by *µ*_Cu_ + *µ*_I_ = *E*(CuI), *µ*_Cu_ ≤ *E*(Cu), and *µ*_I_ ≤ *E*(I), where *E*(Cu), *E*(I), and *E*(CuI) are the total energies of bulk Cu, I, and CuI, respectively.

The chemical potentials are given by *µ*_Cu_ = *E*(Cu) and *µ*_I_ = *E*(CuI) − *E*(Cu) under the Cu‐rich limit, and *µ*_I_ = *E*(I) and *µ*_Cu_ = *E*(CuI) − *E*(I) under the I‐rich limit, respectively.

The formation energies of I‐defect (*E*_form_(I‐defect)) and Cl‐substitution (*E*_form_(Cl‐subst)) on the surface by HDA were estimated by follows.

(5) $$E_{form}\left(I-defect\right)=\left[E\left(CuI-V_{I}\right)+E\left(HDA-I\right)\right]-\left[E\left(CuI\right)+E\left(HDA\right)\right]$$

(6) $$E_{form}\left(Cl-subst\right)=\left[E\left(CuI-Cl\right)+E\left(HDA-I\right)\right]-\left[E\left(CuI\right)+E\left(HDA-Cl\right)\right]$$

where, *E*(CuI), *E*(CuI‐*V*_I_), *E*(CuI‐Cl), *E*(HDA‐I), and *E*(HDA‐Cl) indicate system energies of CuI, CuI with an I vacancy, CuI with a Cl substitution, HDA and I complex, and HDA and Cl complex, respectively. Here, the protonated HDA was used due to its high pKa value (10.6), and anion forms of I and Cl were used for the complex with HDA.

### Raman Measurements

For investigation of SERS performance, R6G was purchased from Sigma–Aldrich Inc. and dissolved in ethanol to prepare solutions with concentrations of 10^−3^ to 10^−6^. R6G solutions were drop‐cast onto 2D Cu plate samples and dried prior to SERS measurements. Raman spectra were collected using a dispersive Raman microscope (ARAMIS, Horiba) equipped with a λ = 532 nm HeNe laser. Gratings with 1800 grooves per 1 mm and an Olympus 40× lens were used. The laser power was 0.2 mW. Raman signals were collected for 3 s for a spectral range between 50–2200 cm^−1^ Raman shift.

## Conflict of Interest

The authors declare no conflict of interest.

## Author Contributions

H.J.H., M.Y.Y., and C.L. contributed equally to this work. M.Y.Y. performed the theoretical calculations and co‐wrote the manuscript under the supervision of W.A.G. H.J.H. conducted in situ TEM experiments, analyzed XRD and XPS data, and co‐wrote the manuscript under the supervision of J.J.C. C.L. synthesized the 2D Cu plates and contributed to manuscript preparation under the supervision of C.C. G.J. carried out the AFM analysis. J.L.H. performed aberration‐corrected STEM imaging and EDX mapping. C.C., J.J.C., and W.A.G. conceived and supervised the project, analyzed the data, and contributed to manuscript writing. All authors discussed the results and approved the final version of the manuscript.

## Supporting information



Supporting Information

Movie S1

Movie S2

Movie S3

## Data Availability

The data that support the findings of this study are available from the corresponding author upon reasonable request.
