# Supplementary material for: Mechanistic Transformation of CuI Nanoparticles Into Oxidation‐Resistant 2D Copper Nanoplates
Source: Small. 2025 Nov 18;22(1):e08098. doi: 10.1002/smll.202508098 (PMC12757985; doi:10.1002/smll.202508098)
Supplement: Supplementary file 1 — Supporting Information [file SMLL-22-e08098-s002.docx]

Supporting Information

Mechanistic Transformation of CuI Nanoparticles into Oxidation-Resistant 2D Copper Nanoplates

*Hyeuk Jin Han^1,2†^, Moon Young Yang^3†^, Changsoo Lee^4†^, Gangtae Jin^1,5^, James L Hart^1^, Rabecca Mutheu^6^, Hyung il Lee^6^, Seo Hyun Kim^7^, Hanhwi Jang^7^, Minjoon Kim^7^, Yeon Sik Jung^7^, William A. Goddard III^3*^, Judy J. Cha^1*^, Chungseok Choi^6*^*

**Supplementary Text**

The main feature of the defective nanoparticle in Figure 3f of the main text is that the CuI (111) spacing of 3.5 Å is reduced to half its nominal value within the defective region, i.e. the lattice spacing is reduced to 1.75 Å. As mentioned in the main text, this observation can be explained by a dislocation with both the line vector ***L*** and the burgers vector ***b*** being in the imaging plane (or at least having a non-zero projection within the imaging plane). To qualitatively show this behavior, Supplementary Figure S4a highlights the defective nanoparticle alongside a STEM simulation of CuI with a dislocation. Away from the dislocation line vector ***L***, the lattice spacing is 3.5 Å, and along ***L*** the observed lattice spacing is 1.75 Å. Thus, the defective nanoparticle shown in Figure 3f of the main text can be explained by a dislocation, although we do not claim that this is a unique solution, and other defects are also possible.

To generate the STEM simulation, we start with the cif file for cubic CuI (mp-22895), and we then construct a spherical supercell with a diameter of 12 nm. We add a screw dislocation running through the center of the nanoparticle with a line vector of ***L*** = [11-1] and a burgers vector ***b*** along [110] (Supplementary Figure S4b). For each atom in the supercell, we find the shortest vector ***v*** which connects atom *i* to the line vector ***L***. Note that all vectors ***v*** are orthogonal to ***L*** and thus lie within (111) plane (Supplementary Figure S4c). We then determine the magnitude of ***v*** as *v* = |***v***| and the angle θ of ***v*** within the (111) plane using the atan2 function. As the vector ***v*** wraps clockwise around ***L***, θ varies from 0 to 2π, with θ = 0 for ***v*** along [112] (Supplementary Figure S4c). Each atom *i* within the supercell is then displaced by the vector **δ** according to:

$$\boldsymbol{\delta}_{\boldsymbol{i}}=\boldsymbol{b}\left( \frac{\theta_{i}}{2\pi} \right)\left( \frac{v_{i}}{1 nm} \right)_{MAX=1}$$

In words, each atom is displaced along ***b***, with the displacement magnitude normalized by two terms. The first term relates to the angular position of the atom. For ***v*** along [112] this term is zero, and ***δ*** = 0. As ***v*** wraps around ***L*** clockwise, this term increases and approaches 1 as θ approaches 2π. The second term relates to the distance of atom *i* from ***L***. For atoms within the dislocation core (*v* = 0 nm), this term is zero and ***δ*** = 0. For atoms further from the dislocation core, δ increases linearly. When *v* = 1 nm, this term saturates at a maximum value of 1. Hence, the maximum value of ***δ*** is the burgers vector ***b***. After generating the dislocation structure, the supercell was rotated off-axis (18° around [11-1]). This dislocation structure is not meant to be rigorously accurate, rather, it is a minimal and simple dislocation structure, meant to qualitatively show how a dislocation looks in STEM.

We used the abtem code to perform the STEM simulation. Given the large size of the supercell, we avoided a full multi-slice simulation. Instead, we generated the atomic potential, integrated along the *z*-axis (the STEM optic axis) and then blurred the image according to the microscope resolution.


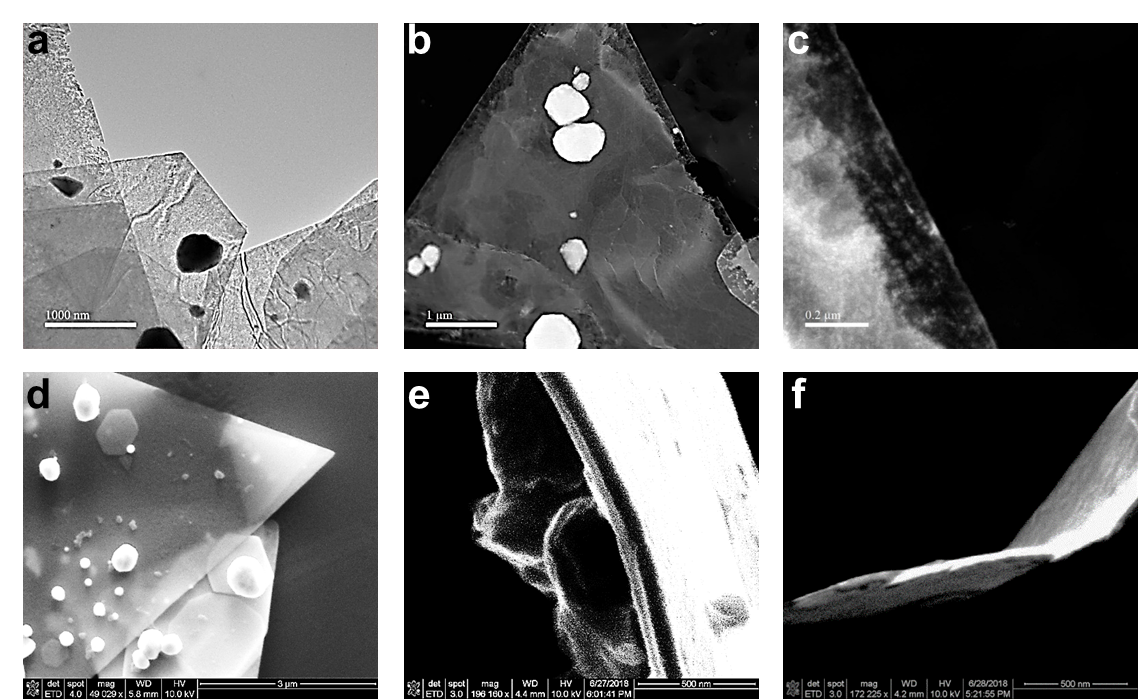


**Figure S1.** **Characterization of 2D Cu plates.** (**a-c**) TEM and STEM images of 2D Cu plates, (**d-f**) SEM images of 2D Cu plates.

**
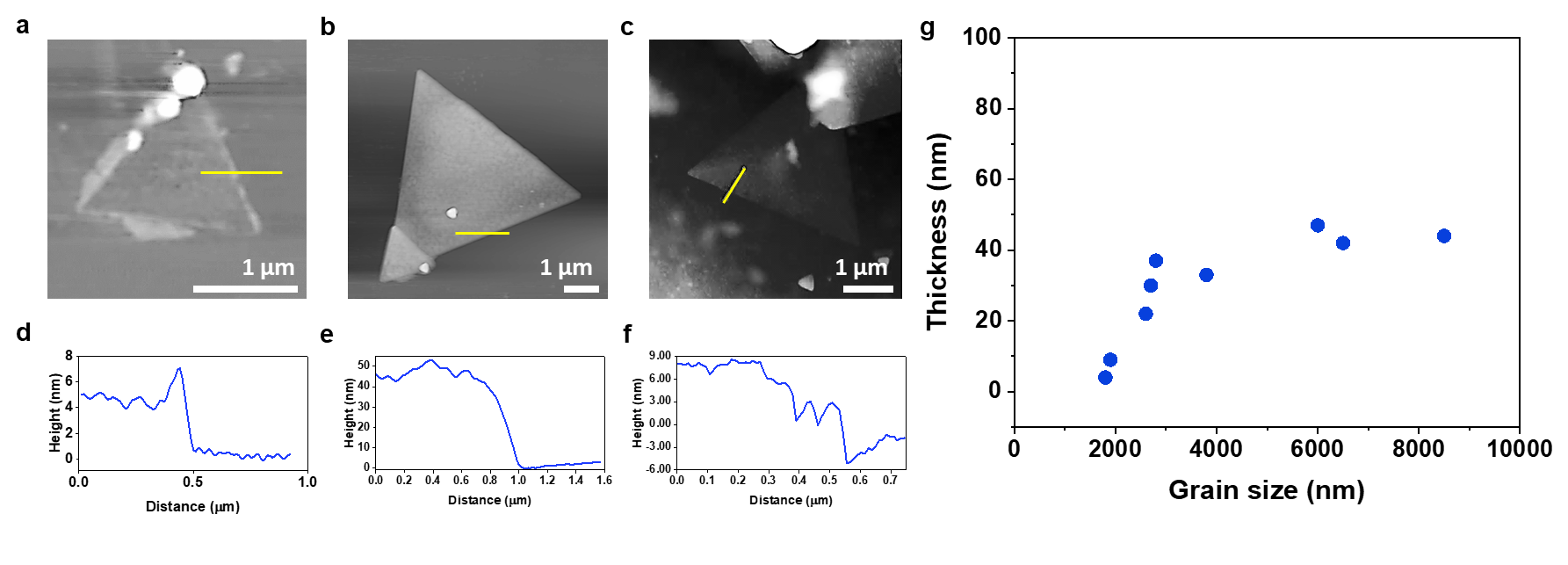
**

**Figure S2. AFM characterizations of a 2D Cu plate.** (a,b,c) AFM topography images of representative 2D Cu plates. (d,e,f) Corresponding height profiles extracted along the yellow lines in (a), (b), and (c), respectively. (g) AFM measured thicknesses of multiple plates with varying lateral sizes.

**
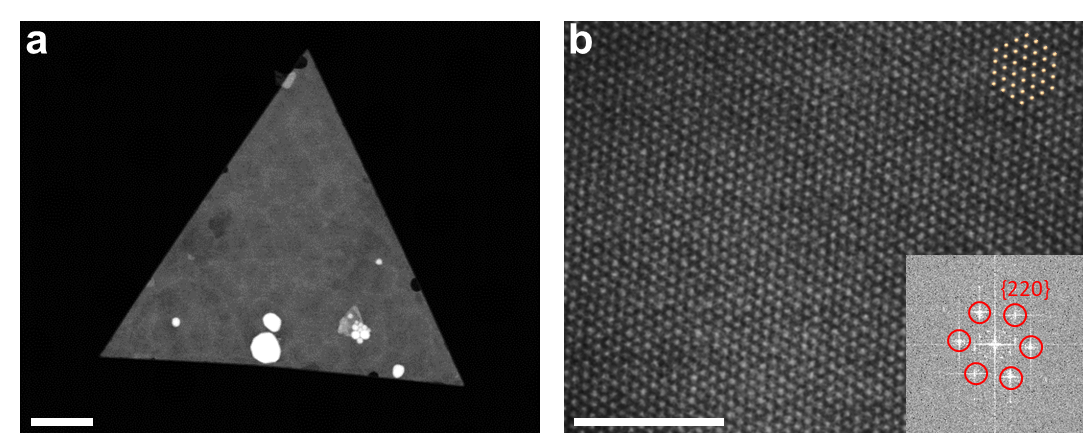
**

**Figure S3.** **STEM image of 2D Cu plate.** (**a**) High-angle annular dark-field (HAADF) STEM image of a 2D Cu plate. Scale bar, 1 μm. (**b**) Atomic-resolution STEM image of a Cu plate showing high crystalline quality observed along the [111] direction, with bright Cu atomic columns. Scale bar, 2 nm. (inset, top) Atomic structure model of Cu viewed along the [111] direction (Yellow: Cu atoms). The inner hexagonal spots in the corresponding FFT pattern are indexed to the {220} family reflections (zone axis [111]).


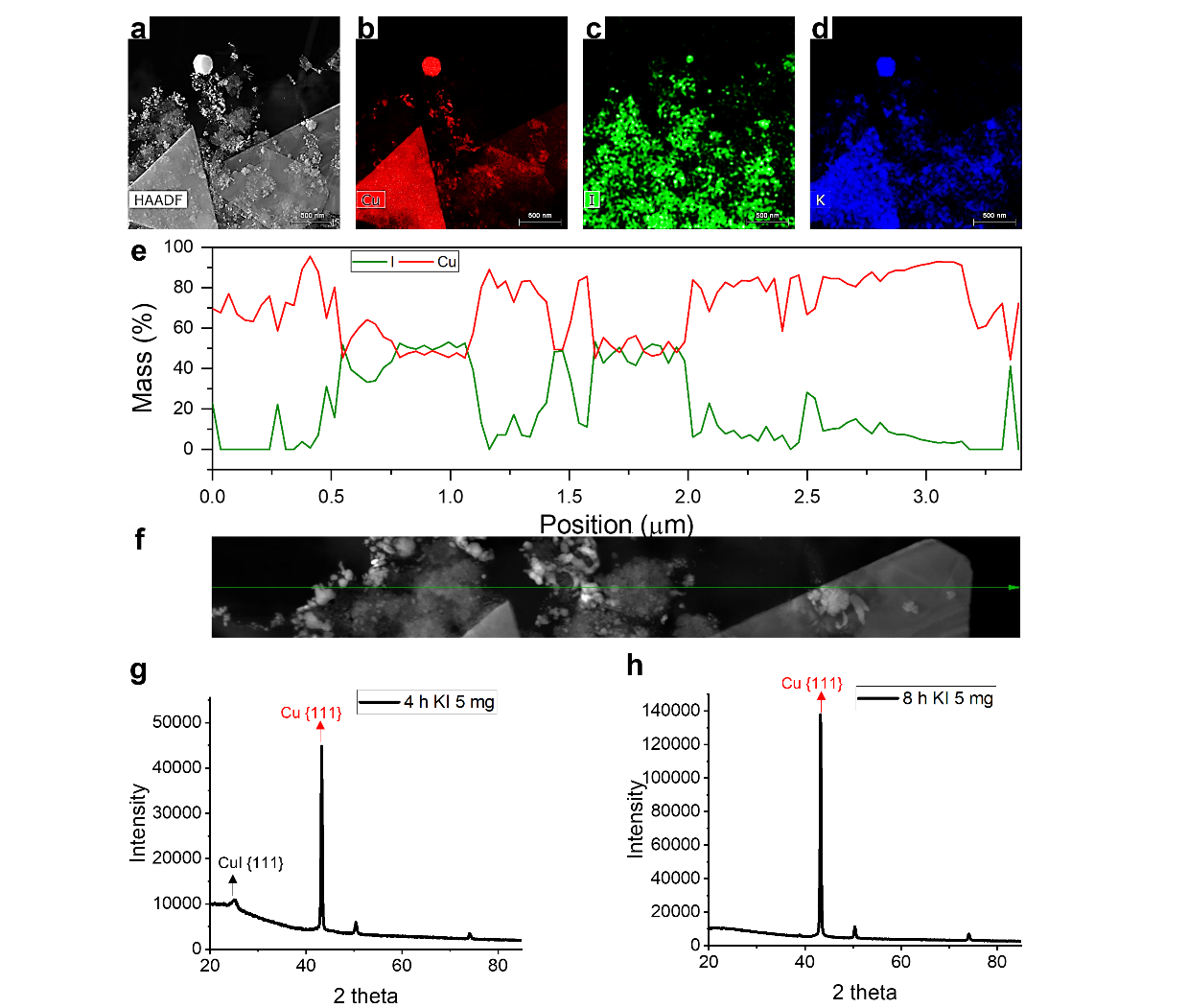


**Figure S4. Synthesis of 2D Cu plate with 5 mg KI in 10 ml water. (a)** HAADF STEM image, (**b**) Cu EDX mapping, (**c**) I EDX mapping, (**d**) K EDX mapping, (**e,f**) EDX line profile, (**g**) PXRD of 2D Cu plates after reaction for 4 h. (**h**) PXRD of 2D Cu plates after reaction for 8 h.

**
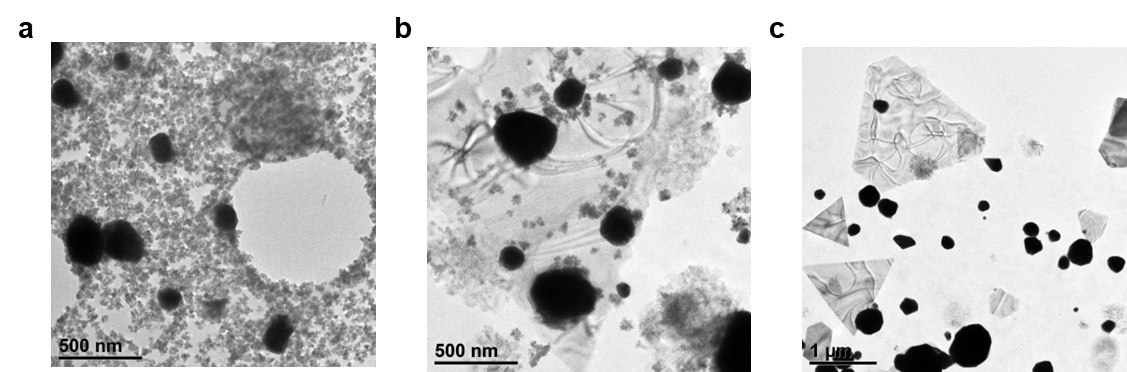
**

**Figure S5. Time-dependent TEM images showing the morphological evolution during the CuI-to-Cu transformation. (a)** CuI nanoparticles observed after 1 h of reaction, exhibiting densely packed spherical morphology. **(b)** Intermediate state after 4 h, showing coexistence of CuI nanoparticles and emerging 2D Cu plates. **(c)** Fully transformed Cu plates obtained after 24 h, with large lateral dimensions and reduced nanoparticle residues.

**
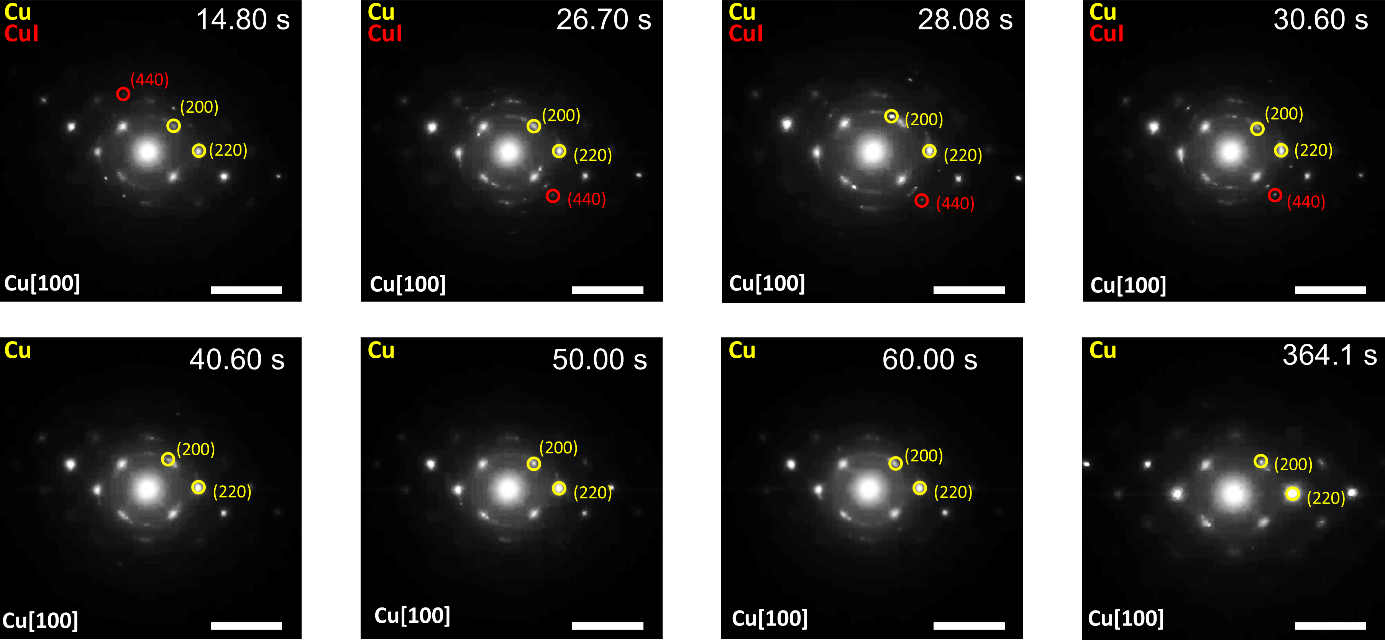
**

**Figure S6.** ***In-Situ* SAED characterization of the CuI-to-Cu phase transformation (corresponding to Supplementary Movie S1).** Scale bar, 5 1/nm.


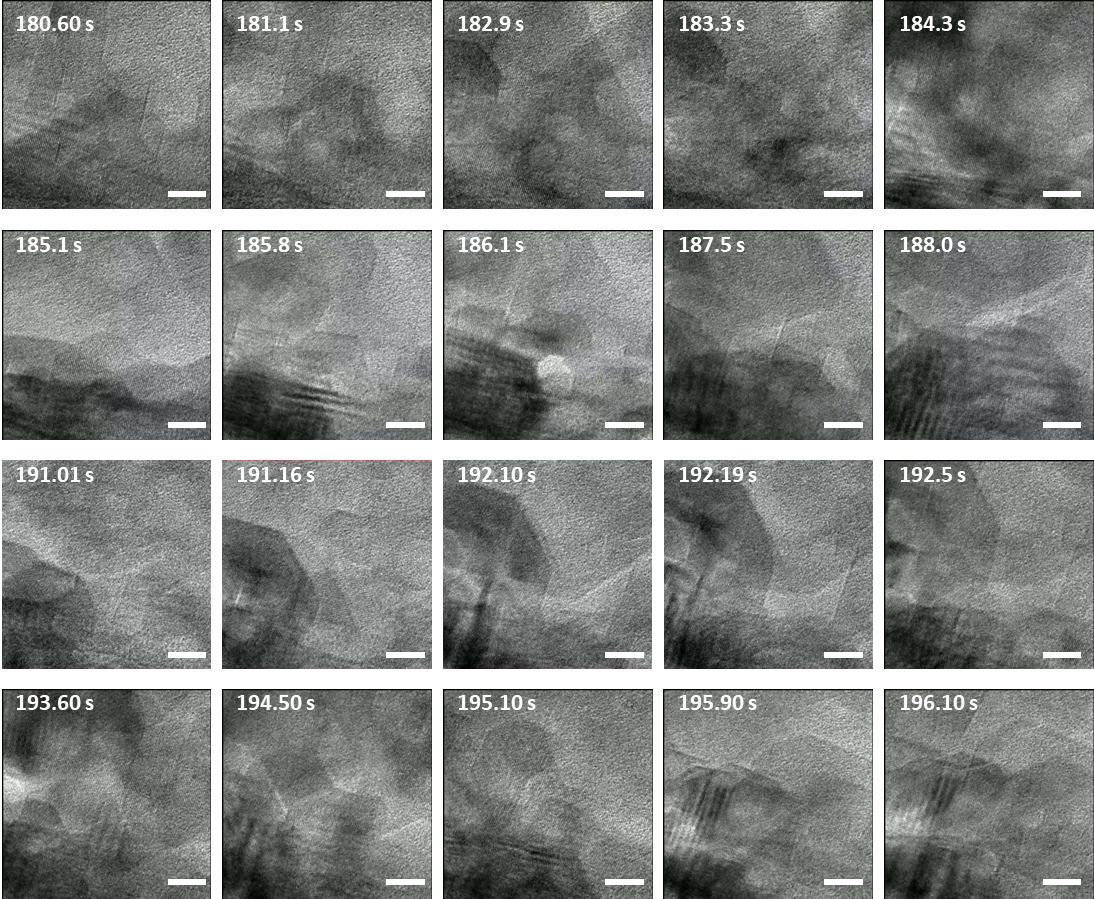


**Figure S7.** **In-situ TEM characterization of the Cu nanosheet transformation (corresponding to Supplementary Movie S2).** Sequential bright-field TEM frames recorded during in-situ liquid-cell heating at 80 °C, showing the real-time morphological evolution to 2D Cu nanosheets. Dynamic timestamps in each frame indicate the elapsed time during the reaction. Scale bars, 5 nm.


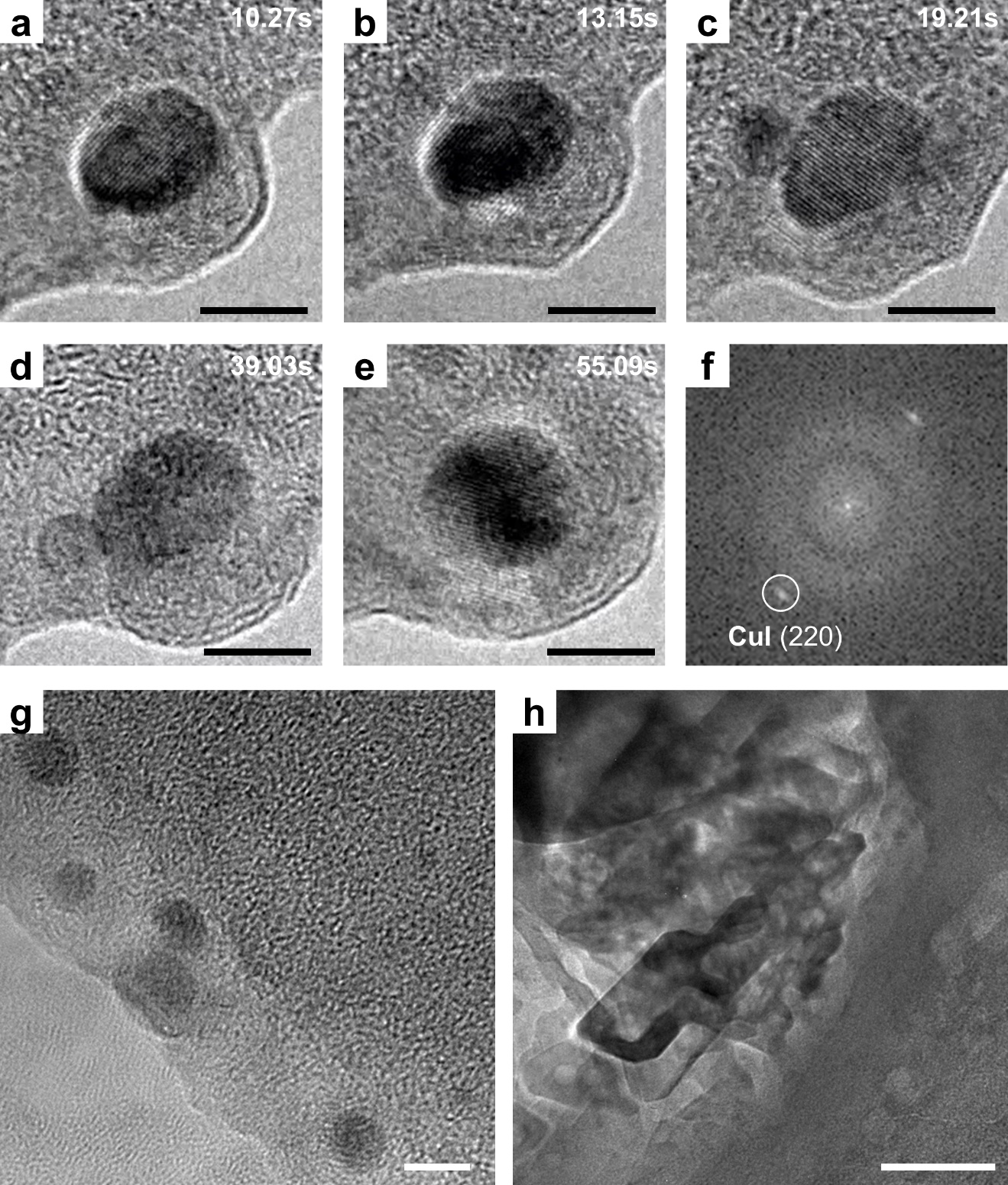


**Figure S8.** ***In situ* TEM characterizations of the CuI NPs.** (**a-e**) Snapshots from an *in situ* movie to examine effects of the e-beam irradiation without heating (Supplementary Movie 3). Scale bars, 5 nm. (**f**) Fast Fourier transform (FFT) of CuI NPs from Supplementary Figure3c. CuI NPs do not transform to Cu plates upon e-beam exposure without heating. (**g**) TEM image of initial CuI NPs in graphene liquid cell before heating. Scale bar, 5 nm. (**h**) TEM image of Cu plate growth in graphene liquid cell after heating. Scale bar, 50 nm.


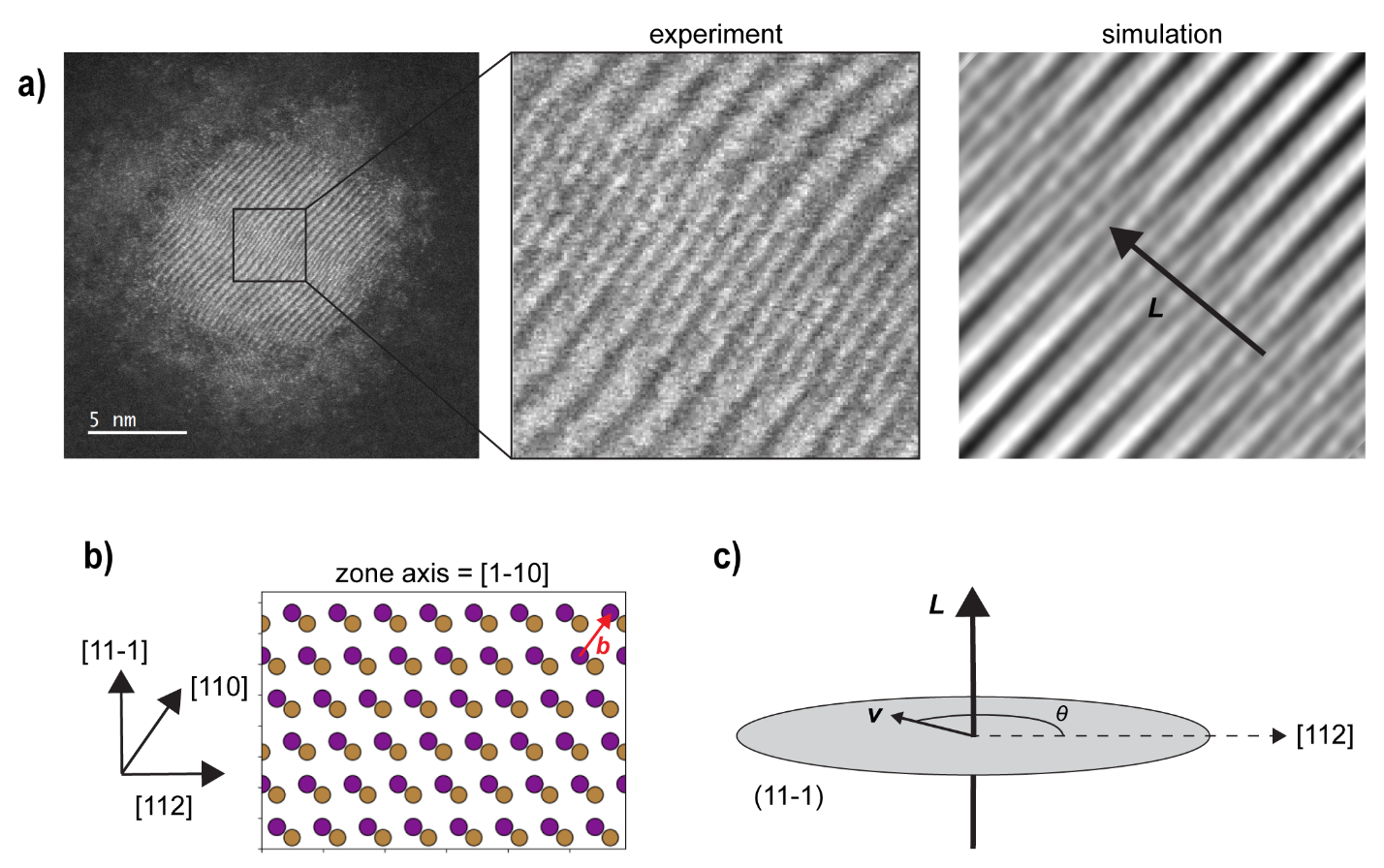


**Figure S9.** (**a**) The same nanoparticle shown in Figure 3f of the main text, highlighting the central defective region, with a STEM simulation for comparison. (**b**) The cubic CuI crystal structure viewed down the [1-10] zone axis. The dislocation line vector ***L*** is along [11-1] and the burgers vector ***b*** is along [110] (shown in red). (**c**) Schematic showing the dislocation line vector ***L***, the (11-1) plane (which is shaded gray), and an example vector ***v***, which marks the position of an atom within the supercell. The angle of ***v*** is measured relative to the [112] vector. See the supplementary text.


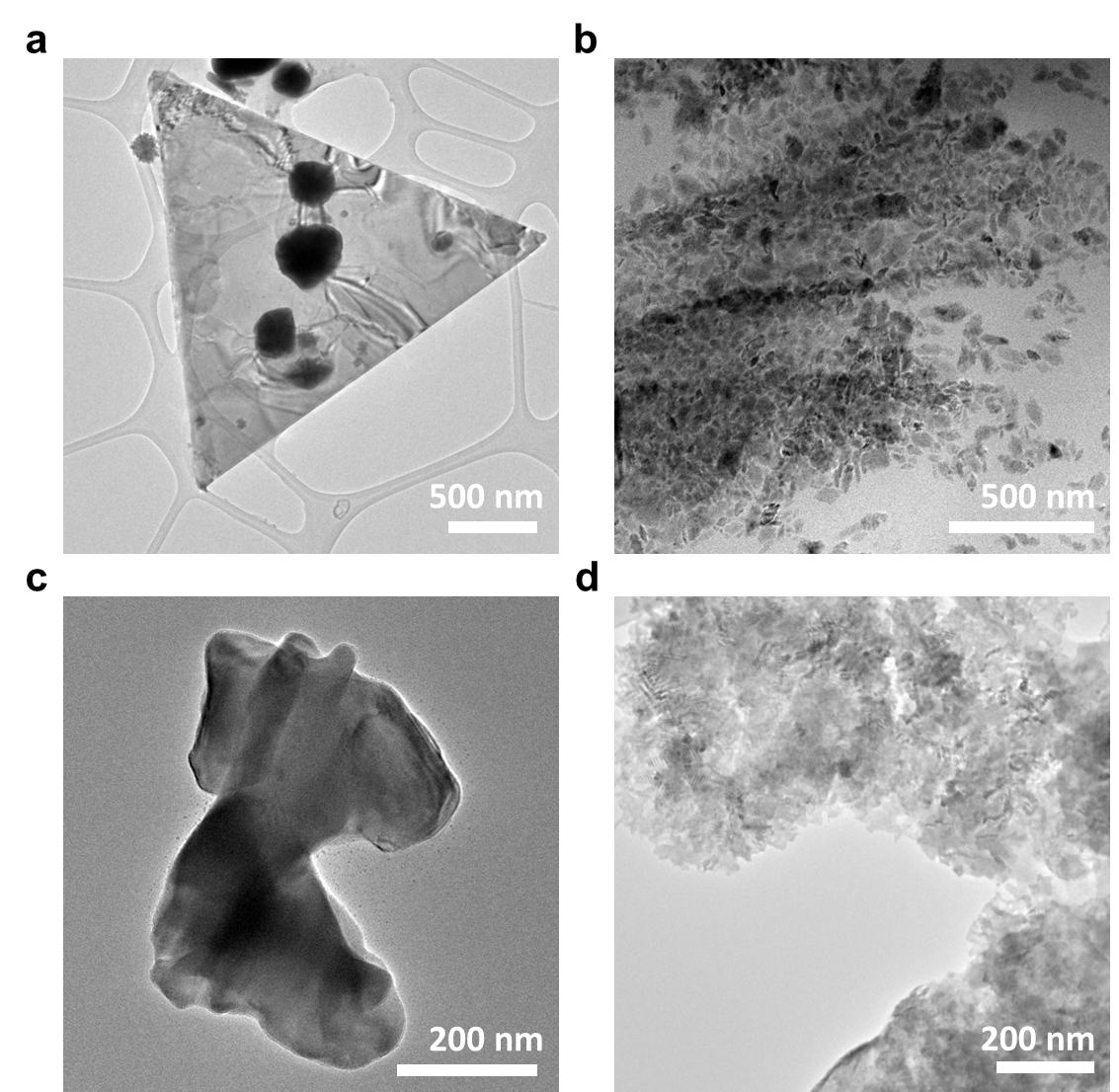


**Figure** **S10. Transformation from commercial CuI NPs to 2D Cu plates at 100 °C for 8 h.** TEM images of synthesized materials prepared with **(a)** all chemical salts; **(b)** without glucose; **(c)** without HDA; **(d)** without KCl.


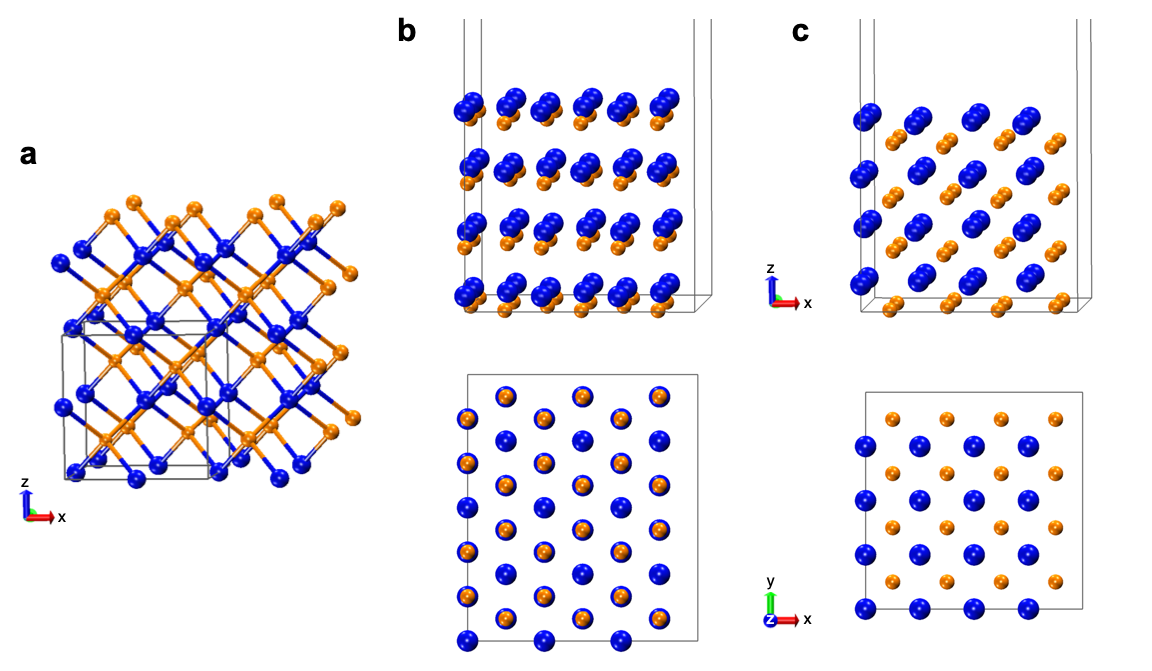


**Figure S11.** **Model structures of γ-CuI.** (**a**) Crystal structure of γ-CuI. Side view (top) and top view (bottom) of (**b**) CuI{111} and (**c**) CuI{100} surface models, respectively. Blue and orange represent Cu and I, respectively, and gray lines indicate the size of the unit cell.

**
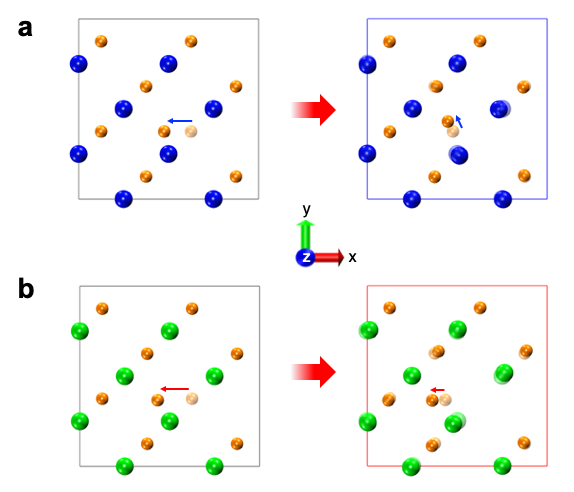
**

**Figure S12. Surface rearrangement of the CuI{100}.**

DFT-predicted surface rearrangement by single Cu atom movement on (**a**) the I-terminated {100} surface and (**b**) the Cl-terminated {100} surface. Here the initial positions are shown with transparency and only top two layers are shown for clarity. Blue, orange, and green represent I, Cu, and Cl, respectively.

**
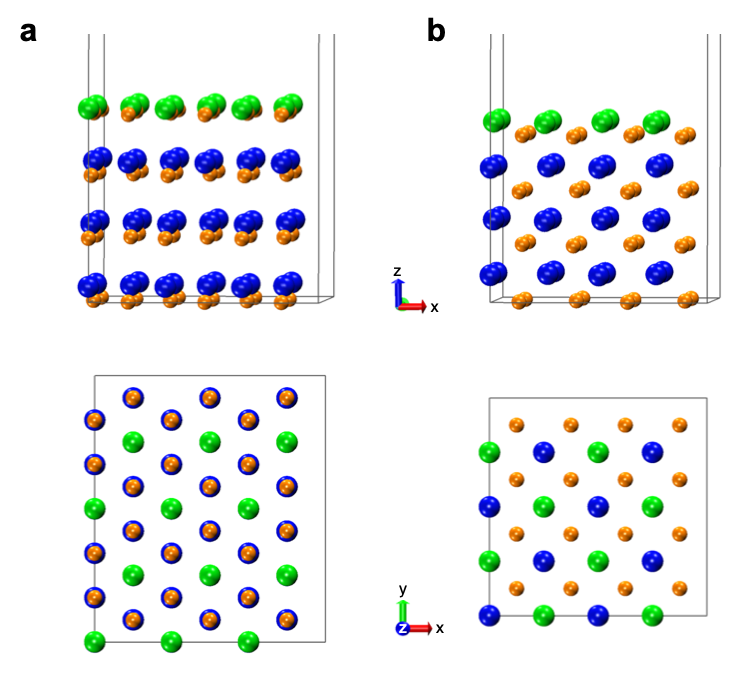
**

**Figure S13.** **Model structures of the Cl-terminated CuI.**

Side view (top) and top view (bottom) of the Cl-terminated (**a**) CuI{111} and (**b**) CuI{100} surfaces, respectively. Blue, orange, and green represent Cu, I, and Cl, respectively, and gray lines indicate the size of the unit cell.

**
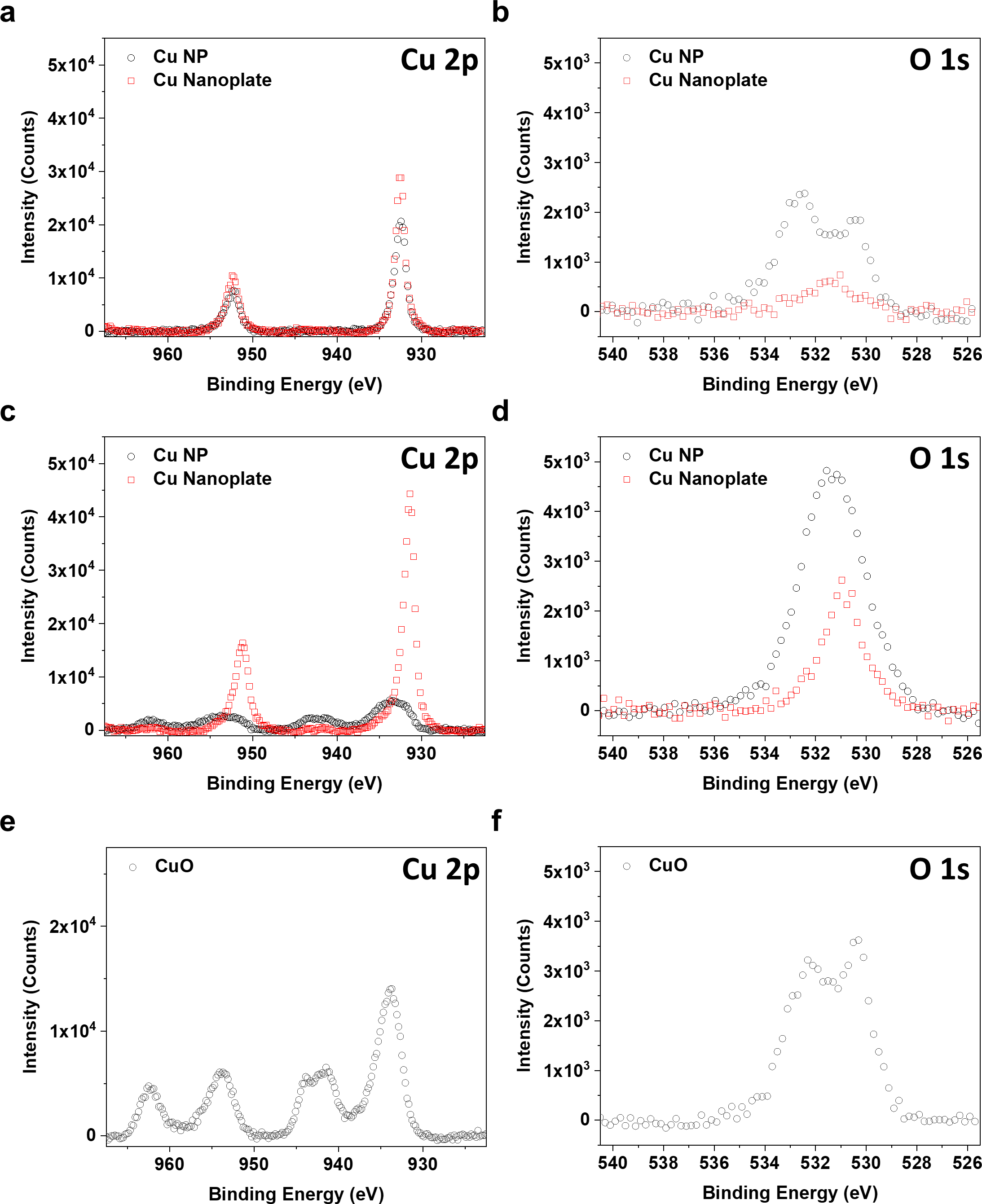
**

**Figure S14. High resolution XPS spectra analysis for oxidation resistance properties of 2D Cu plates.**

The XPS spectra of the commercial Cu NPs and Cu plates at 0 day (**a, b**) and after 105 days (**c, d**) show the Cu 2p (**a, c**) and O 1s (**b, d**) peaks for the composition analysis, respectively. (**e, f**) Bulk CuO was used for reference.

**
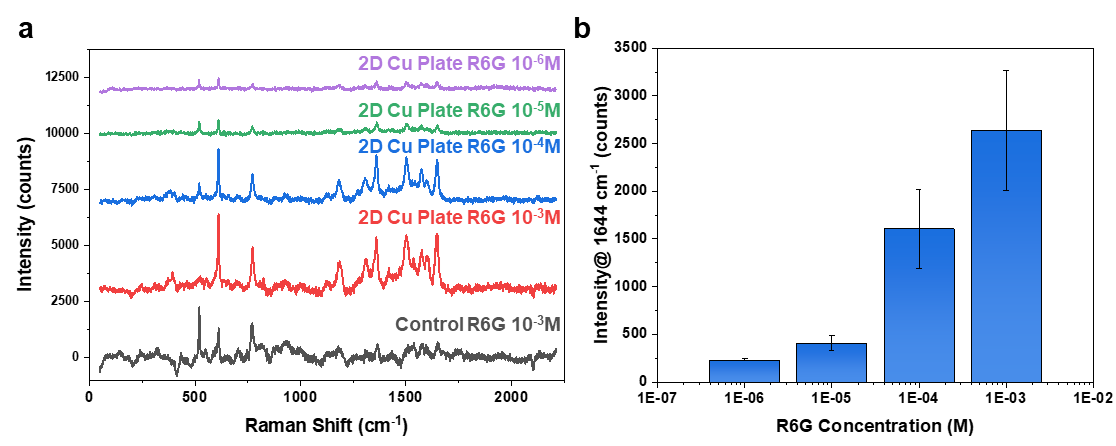
**

**Figure S15.** Raman spectra and quantification of R6G adsorption on 2D Cu plates.
(a) Raman spectra of Rhodamine 6G (R6G) at different concentrations (10^-3^–10^-6^ M) adsorbed on 2D Cu plates, compared with the control (R6G 10^-3^ M without a Cu plate on a Si substrate). Characteristic R6G peaks become increasingly pronounced in the presence of 2D Cu plates, confirming strong surface-enhanced Raman scattering (SERS) activity. (b) Integrated Raman intensity at 1644 cm^-1^ as a function of R6G concentration, showing a monotonic increase in signal intensity with higher R6G concentration, demonstrating the sensitivity and enhancement capability of the 2D Cu plates for molecular detection.

**Table. S1. The calculated CuI surface energies (eV/Å^2^).**

|  | Cu-rich | I-rich |
| --- | --- | --- |
| Cu-terminated CuI{111} | 0.116 | 0.144 |
| I-terminated CuI{111} | 0.021 | -0.007 |
| Cu-terminated CuI{100} | 0.112 | 0.136 |
| I-terminated CuI{100} | 0.014 | -0.010 |

**References**

1. Helander, M. G. et al. Pitfalls in measuring work function using photoelectron spectroscopy. *Appl. Surf. Sci.* **256**, 2602–2605 (2010).
2. Han, H. J. et al. Unconventional grain growth suppression in oxygen-rich metal oxide nanoribbons. *Sci. Adv.* **7**, eabh2012 (2021).
3. Kresse, G. et al. Ab initio molecular-dynamics simulation of the liquid-metal-amorphous-semiconductor transition in germanium. *Phys. Rev. B Condens. Matter* **49**, 14251–14269 (1994).
4. Perdew, J. et al. Generalized gradient approximation made simple. *Phys. Rev. Lett.* **77**, 3865–3868 (1996).
5. Johnson, E. R. & Becke, A. D. A post-Hartree-Fock model of intermolecular interactions: Inclusion of higher-order corrections. *J. Chem. Phys.* **124**, 174104 (2006).
6. Henkelman, G. et al. A climbing image nudged elastic band method for finding saddle points and minimum energy paths. *J. Chem. Phys.* **113**, 9901–9904 (2000).
7. Sundararaman, R. & Goddard III, W. A. The charge-asymmetric nonlocally determined local-electric (CANDLE) solvation model. *J. Chem. Phys.* **142**, 064107 (2015).
8. Sundararaman, R. et al. JDFTx: Software for joint density-functional theory. *SoftwareX* **6**, 278–284 (2017).
9. Zhu, J. et al. Stability and electronic properties of polar and non-polar surfaces of CuI. *Appl. Surf. Sci.* **268**, 87–91 (2013).
